# ATPase Site Architecture Is Required for Self-Assembly and Remodeling Activity of a Hexameric AAA+ Transcriptional Activator

**DOI:** 10.1016/j.molcel.2012.06.012

**Published:** 2012-08-10

**Authors:** Nicolas Joly, Nan Zhang, Martin Buck

**Affiliations:** 1 Division of Biology, Sir Alexander Fleming Building, Imperial College London, Exhibition Road, London SW7 2AZ, UK

## Abstract

AAA+ proteins (ATPases *a*ssociated with various cellular *a*ctivities) are oligomeric ATPases that use ATP hydrolysis to remodel their substrates. By similarity with GTPases, a dynamic organization of the nucleotide-binding pockets between ATPase protomers is proposed to regulate functionality. Using the transcription activator PspF as an AAA+ model, we investigated contributions of conserved residues for roles in ATP hydrolysis and intersubunit communication. We determined the R-finger residue and revealed that it resides in a conserved “R-hand” motif (R_x_D_xxx_R) needed for its “*trans*-acting” activity. Further, a divergent Walker A glutamic acid residue acts synergistically with a tyrosine residue to function in ADP-dependent subunit-subunit coordination, forming the “ADP-switch” motif. Another glutamic acid controls hexamer formation in the presence of nucleotides. Together, these results lead to a “residue-nucleotide” interaction map upon which to base AAA+ core regulation.

## Highlights

► Communication between Walker B and *trans*-acting residues ► Essential role of the divergent Walker A glutamate ► An R-finger as part of an R-hand ► Subunit-subunit coordination by ADP in an AAA+ protein, the ADP-switch

## Introduction

AAA+ proteins (ATPases *a*ssociated with various cellular *a*ctivities) are present in each kingdom of life. In spite of high diversity of substrates and differences in their precise organization, these proteins all use nucleotide binding and hydrolysis to achieve their function. The nucleotide binding pocket formed at the interface between two adjacent subunits is consistent with the observation that AAA+ ATPases oligomerize (usually in hexamers) for activity. This configuration may support nucleotide-driven motions and amplification of conformational changes within the hexamer through protomer-protomer communication.

AAA+ ATPase family members are defined by common structural and functional motifs, including Walker A and B motifs and the second region of homology (SRH) (Wendler et al., 2012). It is generally assumed that the Walker A (consensus sequence G_xxxx_GK [T/S], where x represents any residue) and Walker B (consensus sequence hhhhDE, where h represents a hydrophobic amino acid) motifs are involved in ATP binding and hydrolysis, respectively (Walker et al., 1982). By analogy with GTPases for which efficient GTP hydrolysis depends on the presence of a *trans* arginine residue (the R-finger) (Ahmadian et al., 1997; Scheffzek et al., 1997), putative conserved *trans*-acting arginine residues have been identified in the AAA+ ATPases in the SRH domain. Nevertheless, their roles have only been studied in a few cases. Substitution of such putative R-finger residues drastically reduces protease activities of HslU, Lon, and FtsH (Besche et al., 2004; Bochtler et al., 2000; Ogura et al., 2004; Song et al., 2000); DNA translocase activities of RuvB (Putnam et al., 2001); transcription activation by NtrC (Rombel et al., 1999); and helicase functions of MCM and Ltag (Greenleaf et al., 2008; Moreau et al., 2007). However, the precise identification and mechanism of action of R-fingers remain unclear for most AAA+ ATPases.

We choose PspF AAA+ domain (PspF_1-275_) as an archetypal AAA+ protein core. PspF is a bacterial enhancer binding protein (bEBP, a σ^54^-dependent transcriptional activator) that activates the transcription of the *psp* regulon crucial for phage shock responses (psp) during phage infection and is involved in bacterial pathogenicity (Joly et al., 2010, 2012). We previously reported that in PspF, complex communication networks based on formation of differential salt bridges are effective in allowing the coordination of subunits within the hexamer and the sensing of nucleotide-bound states (Joly and Buck, 2010).

**Figure 1:**
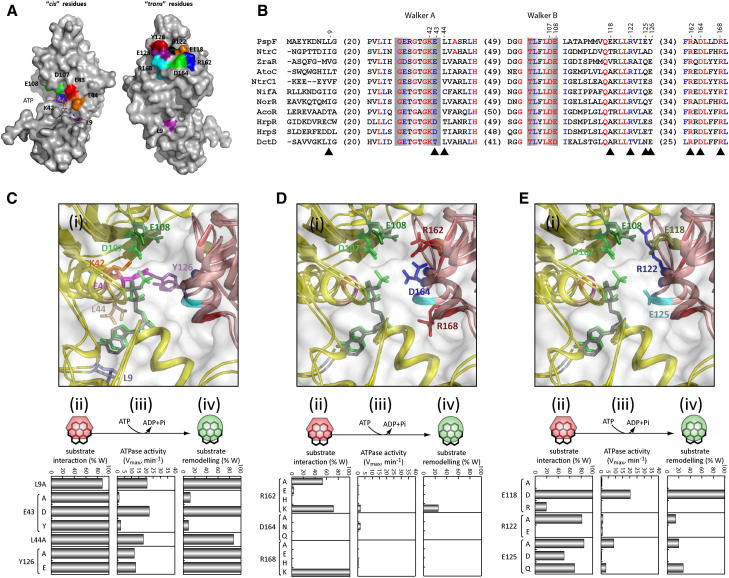
Sequence Alignment, Localization, and Functional Activities of PspF Variants (A) Localization of “*cis*” and “*trans*” residues on the PspF structure (pdb 2C9C) (Rappas et al., 2006). Figure prepared using PyMol software. (B) Sequence alignment of PspF from *Escherichia coli*, NtrC from *E. coli*, ZraR from *E. coli*, AtoC from *E. coli*, NtrC1 from *Aquifex aeolicus*, NifA from *Sinorhizobium meliloti*, NorR from *E. coli*, AcoR from *Pseudomonas aeruginosa*, HrpR and HrpS from *Pseudomonas syringae pv. tomato str. DC3000*, and DctD from *Rhodobacter capsulatus*. Numbering is based on PspF sequence. Black triangles: amino acid substitution of the variants studied. (C–E) Side-chain orientation in the presence of ATP or ADP (i), substrate interaction activity by quantifying the amount of stable σ^54^-PspF complex formed in the presence of ADP-AlF (ii), ATPase activity (iii) (see Table 1 for detail), and substrate remodeling activity by using RP_o_ formation assay (iv) were tested for L9, E43, L44, and Y126 variants (C); R162, D164, and R168 variants (D); and E118, R122, and E125 variants (E). Experiments were performed at least in triplicate, and the maximal variation observed was lower than 10%.

Here we address the contribution of *cis-* and *trans-*acting residues located within PspF subunit interface to the optimal nucleotide dependent activities. For clarity, hereafter we refer to “*cis* residues” when the residues are located in the same subunit as the Walker A and B motifs and “*trans* residues” when the residues are contained in the adjacent subunit directly facing the Walker A and B motifs. From structural analyses and sequence alignments (Figure 1), we identified key candidate residues that can be divided into three major classes: (1) Walker A motif related, (2) potential R-finger residues, and (3) *trans* charged residues. We characterized the unexpected role of the divergent Walker A residue (E43) and its contribution to signal coupling and intersubunit communication (*^cis^*E43-*^trans^*Y126 pair). We also determined that the PspF R-finger residue is *^trans^*R162, revealing a new “R-hand motif” (*^trans^*R162-*^trans^*D164-*^trans^*R168) and a complex communication network at the level of the nucleotide binding pocket. These findings allow us to propose a “residue-nucleotide” interaction map upon which to base AAA+ core regulation.

## Results

### The Walker A Motif Is Involved in an Unexpected *trans* Subunit Communication Pathway

In PspF the Walker A motif, essential for nucleotide binding, is G_xxxx_GKEL (versus consensus G_xxxx_GK[T/S]) (Walker et al., 1982). Schumacher et al. (2004) have shown that substitution of the Walker A lysine (K42A) negatively affects ATP binding and all the related protein activities, but the role(s) of the adjacent residues (E43 and L44) have not been investigated (Figure 1A). Strikingly, the PspF E43 differs from the typical Walker A consensus sequence (T/S) in ATPase, but is nevertheless conserved within bEBPs (the AAA+ Clade 6), suggesting a specific role in the regulation of these protein activities. Structural data suggest that the side chain of E43 is in close proximity to that of *^trans^*Y126 (present in adjacent subunit) when bound to ADP, suggesting a nucleotide-dependent function of this pairwise interaction. Analysis of the structural data of the nucleotide ribose region also suggests a role of residues L44 and L9 in stabilization of nucleotide binding.

Substitutions of E43 (E43A and E43Y) affect V_max_ and K_M_, whereas the most conservative E43D substitution only reduces V_max_, demonstrating the importance of this charged residue in PspF ATPase activity (Figure 1C and Table 1). We note that the E43 substitutions do not affect PspF binding interaction with its target σ^54^ or substrate remodeling activities, suggesting that E43 is only required for ATP hydrolysis. Substitutions of Y126 (the proposed E43 interaction partner) also affect the ATPase activity, but not σ^54^ interaction or substrate remodeling activities, suggesting that both residues are acting in a similar way. Interestingly, we observed that the Y126 substitutions favored the remodeling activity, using only 30% of WT ATPase activity to perform 100% of WT remodeling activity (similar to the result observed with E43D), indicating a strong retention of energy coupling (Figure 1C).

The alanine substitutions of L9 and L44 similarly change the ATPase activities (50% of WT) with different K_M_ values (2,300 μM and 310 μM, respectively), suggesting a differential contribution of these residues to nucleotide binding versus hydrolysis (Figure 1C). Strikingly, their σ^54^ interaction or transcription activation activities were not greatly affected, suggesting that both L9 and L44 are only contributing to “optimization” of nucleotide hydrolysis to some extent by interacting with the ribose sugar to influence the K_M_ (Table 1).

We propose that the E43-Y126 “pair interaction” might have an important negative regulatory function in the WT protein to limit the amount of ATPase-driven remodeling. In addition, L9 could have a direct role in stabilization of the bound nucleotide via its interaction with the 2′OH of the ribose. L44 could have a more direct role in catalysis rather than in nucleotide binding, possibly by positioning the β-γ phosphates via its interaction with the base of the nucleotide.

### Conserved Arginines Proposed to Be the Putative R-Finger

For PspF there is no definitive identification of the R-finger residue. Zhang et al. (2002) have proposed that R162 could be PspF's R-finger by analogy with NtrC R294. Later, Schumacher et al. (2004) suggested that the PspF R-finger could be R168, based on the nucleotide-independent oligomerization property observed with R168A. In this study, we identified an additional residue, D164, located in α helix 6 between R162 and R168 (Figure 1A), with its side chain pointing in the direction of the nucleotide and adopting different conformations dependent on the nucleotide bound (Figure 1D). To evaluate the role of R162, D164, and R168, we substituted each residue and tested their influence on protein activities.

Substitutions of R162, D164, and R168 drastically reduce the PspF ATPase activity and yield constitutive hexamers (Figure S1), but differentially affect the σ^54^ interaction. The R168K maintains the full ability to stably interact with σ^54^ (in the presence of ADP-AlF), whereas R162A and R162K exhibit a reduced but significant interaction (while the other substitutions all fail to form this stable complex). Strikingly, the remaining σ^54^ interaction is not productive except for R162K, for which substrate remodeling activity was still observed, suggesting that the polar basic residue (K) at this location is essential to PspF activities. Interestingly, the K_M_ values of R162A, D164A, and R162K are lower than that of WT (90 μM, 120 μM, and 200 μM versus 250 μM, respectively), whereas all the R168 variants tested exhibit a K_M_ higher than 3,000 μM. We now propose that residues R162 and R168 play different roles in nucleotide binding (Table 1).

We conclude that the R-finger residue—previously defined as a polar basic residue important for nucleotide stabilization and *trans* communication at the interface between two adjacent subunits—in PspF is R162. Although residue R168 has a clear contribution to the nucleotide-dependent PspF activity, it has a more pronounced impact on the final remodeling event than does residue R162. Overall, instead of identifying a single dominant R-finger residue in PspF, we revealed the existence of three highly conserved residues (located in α helix 6) that all play a major role in *trans* subunit stabilization during nucleotide binding and catalysis. We now propose that the previously described R-finger in bEBPs is more likely to be an “R-motif” (or “R-hand”) with a consensus sequence of R_x_D_xxx_R.

### In *trans* Communication at the Level of Nucleotide Binding Pocket

In AAA+ ATPases, the nucleotide-dependent motions used for substrate binding and remodeling are highly regulated. Therefore, we investigated the potential contribution of three newly identified charged residues (E118, R122, and E125) located in the nucleotide binding pocket as potential *trans*-acting residues (Figure 1A).

**Figure 2:**
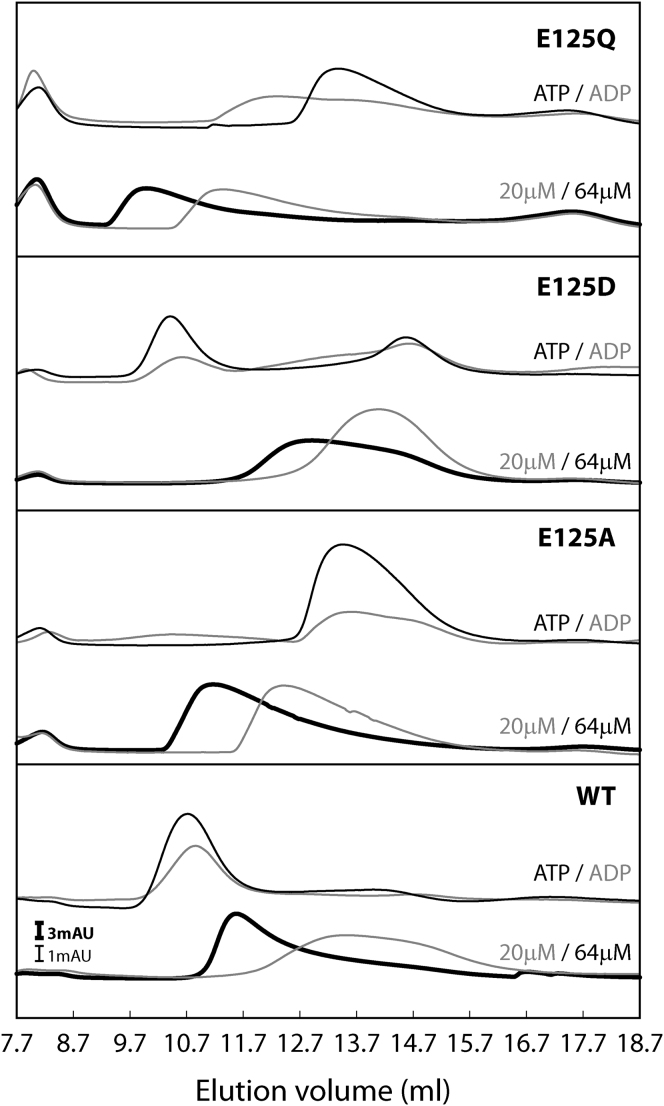
The Presence of Nucleotide ATP or ADP Inhibits E125 Variant Hexamer Formation We compared the elution profile obtained for E125 variants and WT in the absence or presence of nucleotide (ATP or ADP). Gel filtration on Superdex 200 was performed at 4°C. The scale bars give the scale of ordinate axis; absorption units (AU) correspond to an A_280nm_ of 1.

Substitution of the nonconserved residue E118 (E118D) slightly compromised the hexamer formation (Figure S1) and ATPase activity (with a 2-fold reduction), but not its ability to interact with σ^54^ or to initiate RP_o_ formation (Figure 1E and Table 1). In contrast, the E118A and E118R substitutions favored constitutive hexamer formation, drastically reduced the ATPase activity and σ^54^ interaction, and completely abolished RP_o_ formation. This observation suggests that the presence of a negative charge at this position is crucial for PspF activities. These results also suggest that E118 is involved not only in nucleotide binding and hydrolysis but also in other nucleotide-dependent activities. Substitution of the conserved R122 by A or E drastically reduced the ATPase activity, but favored constitutive hexamer formation (Figure S1) without affecting the K_M_ (Table 1), strongly suggesting that this residue takes an active part in the catalytic reaction during ATP hydrolysis. Interestingly, the R122A was still able to bind stably to σ^54^ in the presence of ADP-AlF where the R122E failed, suggesting that R122 does not directly participate in the nucleotide-driven motions that lead to σ^54^ interaction (but inhibit such conformational changes when the charge is inverted [R122E]). As expected, the RP_o_ formation by R122E was drastically reduced, as R122E was unable to engage σ^54^. Substitutions of the nonconserved E125 by A, D, and Q drastically reduced the ATPase activities and altered the properties of hexamer formation (Figure 2 and Figure S1). Depending on the nature of substitution, E125A forms WT-like hexamers, E125D is defective for hexamer formation, and E125Q favors hexamer formation (see above). Surprisingly, the most conserved substitution, E125D, exhibits the most severe loss of function (with less than 1% of WT ATPase activity), whereas the E125Q allows about 10% of WT ATPase activity, suggesting that the size of the side chain is more important than its charge for activity at position 125 (Figure 1E and Table 1). The σ^54^ interaction of these variants in the presence of ADP-AlF was slightly reduced, but their RP_c_ activating activities were more drastically affected (with no RP_o_ detected for E125D). These data suggest that residue E125 is directly involved in catalysis, but not in substrate (RP_c_) interaction process.

Strikingly, we observed that in the case of E125A and E125Q, oligomer formation is diminished in the presence of ATP or ADP (Figure 2). For the first time, we identified a PspF substitution with which nucleotide binding disfavors oligomer formation. We propose that a crucial role in nucleotide-dependent control of oligomerization must be played by residue E125 (Figure S1).

### In *trans* Complementation of Walker B Defective Variants

**Figure 3:**
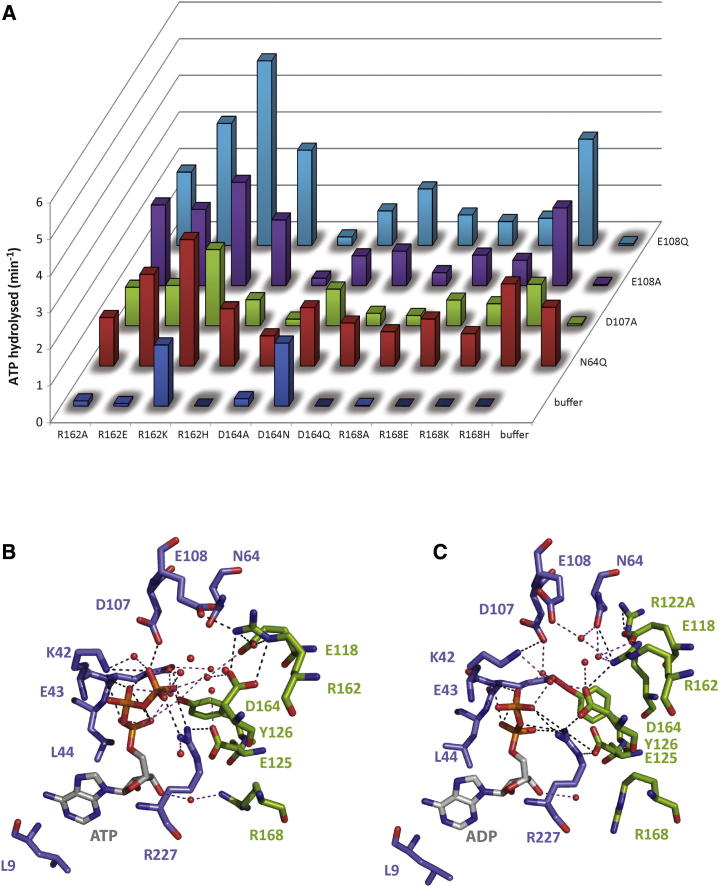
A Complex Interaction Network Is Occurring at the Level of the Nucleotide and PspF Interface (A) ATPase activity of the mixed *cis* (N64, D107, and E108) and *trans* (R162, D164, and R168) defective variants. Histograms represent the k_cat_ observed for the different variants mixed at equimolar concentration. Experiment was performed three times independently, and the maximal error observed was below 10%. (B and C) Schematic of PspF-ATP (B, pdb 2C9C) and PspF-ADP (C, pdb 2C98) interaction networks. Dashed black lanes represent polar contact between side chains, and purple dashed lanes represent polar contact with the solvent. Note that R122 side chain is not resolved in the ATP soaked crystal. Figure was generated using PyMol software.

We investigated further the contribution of interface residues implicated in nucleotide-driven motions using an in vitro complementation approach. This experiment consists of mixing an equal concentration of two defective variants and observing whether the mixing has restored some ATPase activities. We recapitulate that the “*cis* residues” are located in the same subunit as Walker A and B motifs and the “*trans* residues” in an adjacent subunit directly facing the Walker A and B motifs. For *cis*-defective subunit, we used the characterized variants N64Q, D107A, E108A, and E108Q—all highly defective for ATPase activity (Joly et al., 2007, 2008; Schumacher et al., 2004). For *trans* variants, we used the defective R162, D164, and R168 as characterized in this study (Figure 3A and Figure S2).

The R162 and R168 variants only complemented the ATPase activity of defective Walker B variants (D107 and E108), but not N64Q. The R162 variants elicited a more pronounced complementation/stimulation. The strongest complementation effect in D164 variants was observed with D164Q. These results demonstrate that R162 has a more important role in catalysis than R168 and are consistent with the idea that residues R162, D164, and R168 all contribute to optimization of Walker B residues in catalysis. As control, we mixed the *cis* variants N64Q, D107A, E108A, or E108Q with other *cis* variants (for example K42A and E43x), and as expected, we did not observe any increase in ATPase activity (Figure S2A).

In the case of the E43-Y126 pair, we were not able to detect complementation in ATPase activity (Figure S2B). Nevertheless, we observed that the ATPase activity was more negatively affected in the presence of D107A and E108A than with N64Q and E108Q, suggesting an important role of Y126 in regulation of ATP hydrolysis (Figure S2B). A similar effect was observed with E118D. The R122 and E125 variants can complement the *cis*-defective subunits and stimulate ATPase activity, demonstrating their crucial role in optimization of this activity.

We conclude from these results that complementation using the *cis* Walker B defective variants is effective for *trans* R122, E125, R162, and R168 residues. However, each complementation might occur at different stages of the nucleotide binding and hydrolysis process.

## Discussion

We investigated the roles of residues located at the interface between two adjacent subunits and established that the R162 residue was the “genuine” R-finger in PspF. We also demonstrated that residues D164 and R168 were critical for in *trans* subunit communication and nucleotide-dependent activities. We propose that in PspF and all bEBPs (Figure 1A), a functional “R_x_D_xxx_R motif” exists. Strikingly, we showed an interconnection between the conserved Walker B motif and other residues for the *trans*-complementation of *cis*-defective activities.

Interestingly, E125 controls the nucleotide-dependent oligomerization. We observed for the first time that oligomerization of a PspF variant (E125A or E125Q) was disfavored in the presence of nucleotide. This unexpected phenotype strongly suggests a far more complex nucleotide-dependent oligomerization control than previously thought.

Finally, we revealed a new residue pair, E43-Y126, which was responsible for the communication of the ADP-bound state between subunits. It is very tempting to propose that this pair of residues could contribute to ADP release after ATP hydrolysis and so acts as an “ADP-switch” (see detailed discussion below).

### R-Finger versus R_x_D_xxx_R Motif

The precise contribution of an R-finger to ATPase activity and nucleotide-dependent remodeling in AAA+ ATPases has only been studied in a very few cases, and any mechanistic conclusions on the role(s) of an R-finger remain unclear. By comparison with the GTPases, the R-finger of an AAA+ ATPase has been defined as a residue acting in *trans* (compared to the subunit containing the Walker A and B motifs), close to the γ-phosphate of ATP, and favoring efficient ATP hydrolysis by stabilizing the ATP transition state (Ogura and Wilkinson, 2001). Often an arginine residue is identified by sequence alignment and homology in the AAA+ ATPase SRH motif. In the case of PspF, we now discriminate between R162 and R168, which were suggested as putative R-finger residues, and establish that R162 is the PspF R-finger residue. Our biochemical analyses reveal that substitution of R162 with K retains the positively charged functional group for contacting the β-γ phosphate, and the resultant R162K variant is active. This is not the case for the R168K variant. Structural data also fully support that R162 is in closer proximity to the γ-phosphate of ATP than is R168, which is closer to the O2′ of the nucleoside (Figure 3B). Importantly, R162 is not the sole residue acting in *trans* that favors nucleotide binding and hydrolysis; rather, an ubiquitous “R_x_D_xxx_R motif” exists among the bEBPs.

### The “ADP-Switch”: E43-Y126 for ADP Sensing?

We characterized the contribution of the Walker A E43 residue (divergent from the consensus T/S sequence but conserved in EBPs) and showed that it undertook the same function as the conserved T residue as present in other clades of the AAA+ family (Gai et al., 2004; Lenzen et al., 1998; Liu et al., 2000; Yu et al., 1998).

In addition, the analysis of structural data obtained by soaking the PspF crystal with ATP or ADP (Rappas et al., 2006) shows a potential interaction between the *cis* E43 and the *trans* Y126 apparently not conserved in AAA+ ATPases or bEBPs. Interestingly, ADP binding induces a structural change that causes the two side chains of the E43-Y126 pair to clash, possibly triggering a cascade of conformational changes that facilitates the ADP release. In addition, we showed that the E43 substitution greatly reduced both the ATPase activity and RP_o_ formation, whereas the Y126 substitutions only reduced the ATPase activity. The E43-Y126 interaction in the presence of ADP possibly functions to increase the ATPase activity of an adjacent subunit where an ATP is bound. When this interaction is lost, the rate of ATP hydrolysis is significantly reduced. We propose that the charge of the E43 side chain could have a direct role in coupling the ATPase activity to substrate remodeling, consistent with the observation that E43D exhibits a 50% reduction in ATPase activity but retains WT-like RP_o_ formation. In conclusion, we propose that the E43-Y126 interaction pair can serve as an “ADP release switch.”

### bEBPs and R-Fingers

The structure of a NtrC1 variant (^NtrC1^E239A, corresponding to ^PspF^E108A) revealed a heptameric assembly bound to ATP (Chen et al., 2010). The authors propose that the ^NtrC1^R299 residue (corresponding to ^PspF^R168 in PspF) could be the R-finger of NtrC1 because of (1) its spatial proximity to ATP and (2) its ATPase minus phenotype. In our study based on the structural and functional data obtained for the hexameric PspF, we established that the R-finger is ^PspF^R162 (corresponding to ^NtrC1^R293 in NtrC1). The overlay of both monomeric structures (PspF and NtrC1) shows a very high level of structural similarity between both proteins with the only slight difference in the side chain orientation of several residues (Figure S3), including ^PspF^R168 (^NtrC1^R299), but similar for ^PspF^R162 (^NtrC1^R293) and ^PspF^D164 (^NtrC1^D295). Nevertheless, these slight differences cannot explain in simple terms the two different R-fingers identified. We propose that the precise organization of the interface between two adjacent subunits of an oligomer is a key element for the identification of R-finger residues. Starting from very similar monomeric structures, when PspF and NtrC1 organize into hexamers and heptamers, respectively, the interfaces between protomers in the two oligomers will adopt inevitably different configurations (Figure S3). Depending on different oligomeric states of PspF and NtrC1, it is then very rational to observe differential contributions of residues located at the level of interface between these two proteins. If the heptameric structure observed for NtrC1 is physiologically relevant, this protein provides a good example of the evolution of deployment of a conserved R_x_D_xxx_R motif using the same residue for a similar overall activity, but with a different order.

### Nucleotide Binding Pocket Network

Nucleotide interactions in AAA+ ATPases are tightly controlled and critically underlie their remodeling functions. Some residues taking part in this regulation are very conserved in all AAA+ ATPases, whereas others will be more specific of a subclade. Such divergence could reflect the different types of substrate targeted and the different number of ATPs used per remodeling transaction. For processivity (for example, in the case of translocase or helicase), the necessary amount of ATP hydrolyzed by an AAA+ ATPase is likely to be higher than that of a simple productive asymmetrical binding interaction between the substrate and the AAA+ ATPase (for example, for transcriptional activator). It seems that the different clades have elaborated from a central ATPase regulatory core of Walker A and B motifs and R-finger residue much specific fine tuning at the level of the subunit interfaces.

## Experimental Procedures

Detailed protocols are given in Supplemental Information.

### Protein Purification

PspF_1-275_ proteins were purified (Joly et al., 2006). σ^54^ and ^32^P end-labeling HMK (heart muscle kinase)-σ^54^ was purified and labeled (Cannon et al., 2000; Wigneshweraraj et al., 2003). *E. coli* core RNAP enzyme was purchased from Epicenter.

### ATPase Activity

Steady-state ATPase assays were performed at 37°C in the presence of a NADH-coupled regeneration system (Nørby, 1988).

### Native Gel Mobility Shift Assays: Sigma 54 Interaction Assay

Gel mobility shift assays were conducted to detect protein-protein complexes as described in Joly et al., 2007, 2008 using labeled DNA or σ^54^ as indicated in legends.

### In Vitro Open Complex Formation and Full-Length Transcription Assays

Open complex formation assays and full-length transcription were performed as in Joly et al., 2007, 2008. Radiolabeled RNA products were measured by PhosphorImager (Fuji Bas-1500) and analyzed using the Aida software.

### Gel Filtration through Superdex 200

PspF_1-275_WT and variants (at different concentrations) were injected onto a Superdex 200 column (10 × 300 mm, 24 ml, GE Healthcare) at 4°C ± nucleotide as in Joly et al., 2006.

## Figures and Tables

**Table 1:** Kinetic Constants for ATP Hydrolysis for PspF_1-275_ WT and Variants

Protein	V_max_ (min^−1^)	K_m_ (μM)^a^
WT	39.51 ± 4.62	250
L9A	20.72 ± 2.55	2,300
K42A	Not detected	Not detected
E43A	1.37 ± 0.10	750
E43D	22.26 ± 4.80	250
E43Y	2.49 ± 0.17	1,250
L44A	18.16 ± 2.42	310
N64Q	1.50 ± 0.10	500
D107A	0.23 ± 0.05	300
E108A	0.15 ± 0.01	100
E108Q	0.07 ± 0.01	100
E118A	0.60 ± 0.05	>1,500
E118D	20.23 ± 3.70	800
E118R	0.31 ± 0.05	50
R122A	1.26 ± 0.03	300
R122E	0.95 ± 0.06	200
E125A	8.86 ± 0.38^b^	2,000
E125D	0.43 ± 0.01	160
E125Q	3.39 ± 0.38^b^	170
Y126A	11.95 ± 1.77	650
Y126E	12.82 ± 1.03	800
R162A	0.18 ± 0.02	90
R162E	0.28 ± 0.01	>3,000
R162K	1.88 ± 0.29	200
R162H	0.08 ± 0.01	3,000
D164A	0.21 ± 0.02	120
D164N	1.83 ± 0.13^c^	155
D164Q	0.13 ± 0.04	>5,000
R168A	0.17 ± 0.03	>4,500
R168E	0.11 ± 0.03	>3,000
R168K	0.12 ± 0.02	>4,000
R168H	0.43 ± 0.06	>5,000

a Michaelis-Menten kinetic constants for WT and mutated PspF_1-275_ variants in the absence of σ^54^. The data were an average of at least three independent experiments. Maximal error is 10%.

b No V_max_, linear with concentration/no plateau.

c Max already at low concentration/no sigmoidal curve.

## Supplemental Information

Document S1. Figures S1–S3, Table S1, and Supplemental Experimental Procedures
