# Supplementary material for: ATPase Site Architecture Is Required for Self-Assembly and Remodeling Activity of a Hexameric AAA+ Transcriptional Activator
Source: Mol Cell. 2012 Aug 10;47(3-8):484–90. doi: 10.1016/j.molcel.2012.06.012 (PMC3419264; doi:10.1016/j.molcel.2012.06.012)
Supplement: Document S1. Figures S1–S3, Table S1, and Supplemental Experimental Procedures [file mmc1.pdf]

Molecular Cell, Volume *47*

Supplemental Information

ATPase Site Architecture Is Required for Self-Assembly and Remodeling Activity of a Hexameric AAA+ Transcriptional Activator

Nicolas Joly, Nan Zhang, and Martin Buck

SUPPLEMENTAL EXPERIMENTAL PROCEDURES

Plasmids

Plasmid pPB1 encodes the AAA+ domain of *E. coli* PspF, called $PspF_{1-275}$ with an amino terminal 6-His tag in $pET28b^+$ (Bordes et al., 2003). Variants of $PspF_{1-275}$ were generated to yield variants: pNJs and pNZs (Supplemental Table 1S), verified by DNA sequencing.

Protein purification

$PspF_{1-275}$ proteins were purified (Joly et al., 2006). σ^{54} and ^{32}P-end labelling HMK (heart muscle kinase)-σ^{54} was purified and labelled (Cannon et al., 2000; Wigneshweraraj et al., 2003). *E. coli* core RNAP enzyme was purchased from Epicentre. Protein concentration was estimated (Lowry et al., 1951).

ATPase activity

Steady-state ATPase assays were performed at 37°C in the presence of a NADH-coupled regeneration system (Norby, 1988) in a 100 µl final volume, in buffer containing final concentrations of: 25 mM Tris-HCl (pH 8.0), 100 mM KCl, 10 mM $MgCl_2$, 1 mM DTT, 1 mM NADH, 10 mM Phosphoenol pyruvate, 10 U/ml Pyruvate Kinase, 20 U/ml Lactate dehydrogenase, ATP (from none to 50 mM) and $PspF_{1-275}$ (from 0 to 20 µM). Mixing experiments were performed using the same condition in the presence of 2 µM or 4 µM of protein when alone, and 2 µM of protein A +2 µM of protein B when protein were mixed.

Native gel mobility shift assays - sigma 54 interaction assay

Gel mobility shift assays were conducted to detect protein-protein complexes. Assays were performed in a 10 µl final volume containing: 10 mM Tris-acetate (pH 8.0), 50 mM potassium acetate, 8 mM magnesium acetate, 0.1 mM DTT, 4 mM ADP, ± NaF (5 mM) ± ^{32}P-HMK-σ^{54} (1 µM). Where required, $PspF_{1-275}$ WT or variant (5 µM) ± $AlCl_3$ (0.4 mM) were added for a further 20 minutes at 37°C. Complexes were analysed on a native 4.5% polyacrylamide gel. Radiolabelled HMK-σ^{54} was measured by PhosphorImager (Fuji Bas-1500) and analysed using the Aida software.

In vitro Open Complex formation and full-length transcription assays

Open complex formation assays were performed in a 10 μl volume containing: 10 mM Tris-acetate (pH 8.0), 50 mM potassium acetate, 8 mM magnesium acetate, 0.1 mM DTT, 4 mM dATP, 0.1 μM core RNAP enzyme, 0.4 μM σ^{54} and 20 nM promoter DNA (supercoiled *S. meliloti nifH* promoter). The mix was pre-incubated at 37°C for 5 minutes and the reaction started by addition of 5 μM of $PspF_{1-275}$ WT or variants and incubated for varying times at 37°C.

Open complex formation was monitored following the synthesis of the transcript (-1 UpGGG+3) started by simultaneous addition of heparin (100 µg/ml), initiating dinucleotide UpG (0.5 mM), GTP (0.01 mM) and 4 µCi [α-^{32}P]GTP for a further 10 minutes. The reaction was stopped by addition of loading buffer and analyzed on a 20% denaturating gel. Full-length transcription was initiated by adding a mix containing 100 µg/ml heparin, 1 mM ATP, CTP, GTP, 0.5 mM UTP and 3 μCi [α-^{32}P]UTP for a further 10 minutes. The reaction was stopped by addition of loading buffer and analyzed on 6% sequencing gels.

Radiolabelled RNA products were measured by PhosphorImager (Fuji Bas-1500) and analysed using the Aida software.

Gel filtration through Superdex 200

$PspF_{1-275}$WT and variants (at different concentrations) were incubated for 5 minutes at 4°C in buffer containing 20 mM Tris-HCl (pH 8.0), 50 mM NaCl and 15 mM $MgCl_2$, ± 0.5 mM ATP or ADP where indicated. 50 µl samples were then injected onto a Superdex 200 column (10 × 300 mm, 24 ml, GE Healthcare) and equilibrated with the sample buffer ± nucleotide. Chromatography was performed at 4°C at a flow rate of 0.5 ml/min and columns were calibrated with globular proteins: thyroglobulin (669 kDa), apoferritin (443 kDa), b-amylase (200 kDa), bovine serum albumin (66 kDa) and carbonic anhydrase (29 kDa). All experiments were repeated at least twice and the elution profiles obtained were similar. Proteins were detected at 280 nm .

β-galacosidase assays

To perform *in vivo*, β-galacosidase assays, variant constructs were sub-cloned from pET28b to pBAD18C using XbaI/HindIII restriction enzyme. After transformation into cells containing a *pspA-lacZ* reporter construct inserted on the chromosome, cells were grown overnight at 37°C in LB broth containing the appropriate antibiotic and then diluted 100-fold (initial OD_{600}≈0.025) into the same medium (5 ml). Following incubation to OD_{600}≈0.30, cultures were induced with arabinose for 1 h, further grown to mid-exponential phase (OD_{600}≈0.5-0.6) and then assayed for β-galactosidase activity as described by (Miller, 1972). Enzyme activities (in Miller units) represent the means ± SD of the triplicate average values from at least six independent cultures.

Table S1, related to Table 1: Plasmids used in this work.

	in pET28b	reference
WT	pPB1	*(Bordes et al., 2003)*
L9A	pNJ38	*this study*
K42A		*(Schumacher et al., 2004)*
E43A	pNJ39	*this study*
E43D	pNJ40	*this study*
E43Y	pNJ41	*this study*
L44A	pNJ42	*this study*
N64Q		*(Joly et al., 2008)*
D107A		*(Schumacher et al., 2004)*
E108A		*(Joly et al., 2007)*
E108Q		*(Joly et al., 2007)*
E118A	pNJ43	*this study*
E118D	pNJ44	*this study*
E118R	pNJ45	*this study*
R122A	pNJ46	*this study*
R122E	pNJ47	*this study*
E125A	pNZ1	*this study*
E125D	pNZ2	*this study*
E125Q	pNZ3	*this study*
Y126A	pNJ48	*this study*
Y126E	pNJ49	*this study*
R162A		*(Schumacher et al., 2004)*
R162E	pNJ50	*this study*
R162H	pNJ51	*this study*
R162K	pNJ52	*this study*
D164A	pNZ4	*this study*
D164N	pNZ5	*this study*
D164Q	pNZ6	*this study*
R168A		*(Schumacher et al., 2004)*
R168E	pNJ53	*this study*
R168H	pNJ54	*this study*
R168K	pNJ55	*this study*

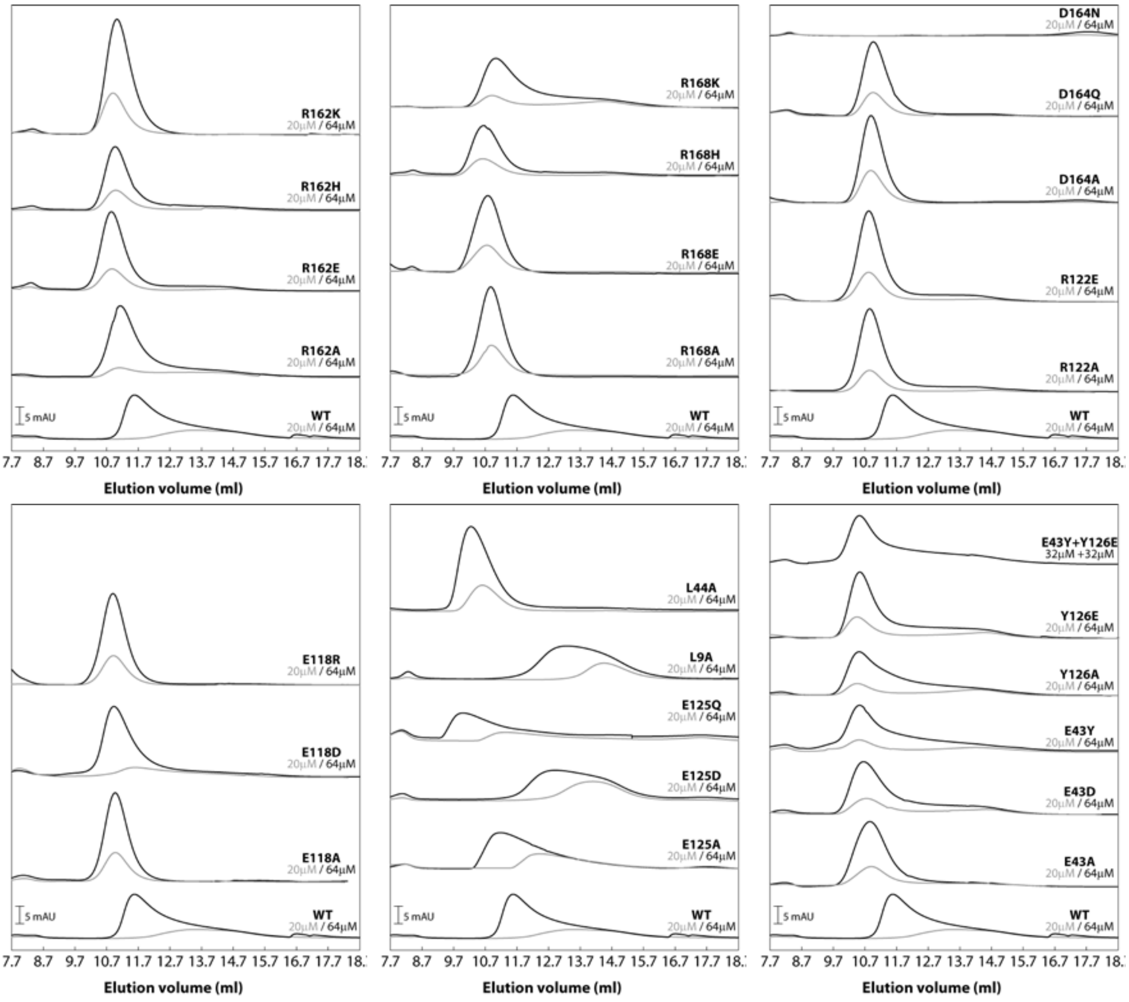

Figure S1, related to Figure 1: Oligomerization of PspF variants. Gel filtration on Superdex 200 was performed at 4°C. The scale bars give the scale of ordinate axis; absorption units (AU) correspond to an A_{280nm} of 1.

Oligomerization is one of the conserved properties of AAA+ proteins needed for the formation of the active catalytic site at the interface between subunits. We observed that the substitution of almost all the residues targeted in this study (except for L9 and E125) have a major effect on the oligomerization, favouring formation of a hexameric state similar to the one observed for the WT in the presence of nucleotide. This outcome is in complete agreement with studies suggesting that the PspF hexamer exists in a conformation non-functional for stable binding σ^{54} in the absence of nucleotide, insured by the complex interaction network between the residues located at the level of the interface (Joly and Buck, 2010). Substitution of the residues taking part in this network will "unlock" the conformation and the hexamer will adopt an "activate conformation". In the case of L9A and E125D, the oligomer formation is reduced compare to WT, but is still dependent on the protein concentration and/or on the presence of nucleotide.

A.

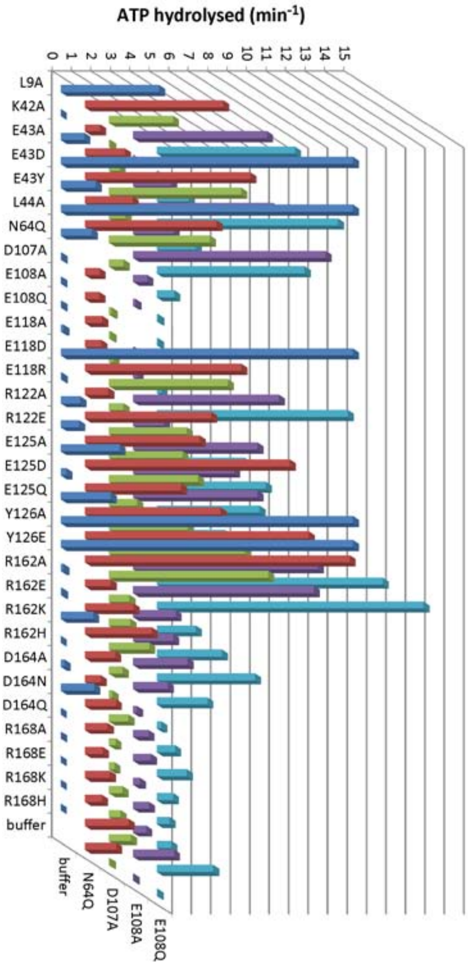

B.

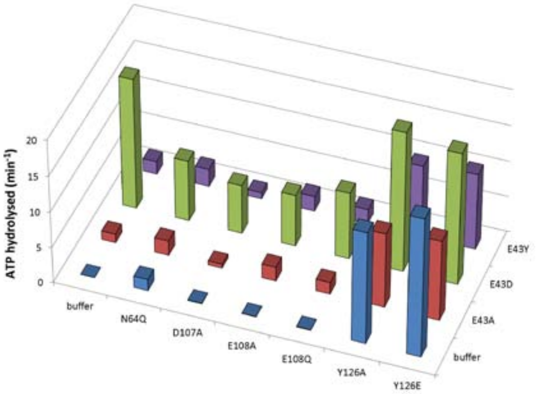

C.

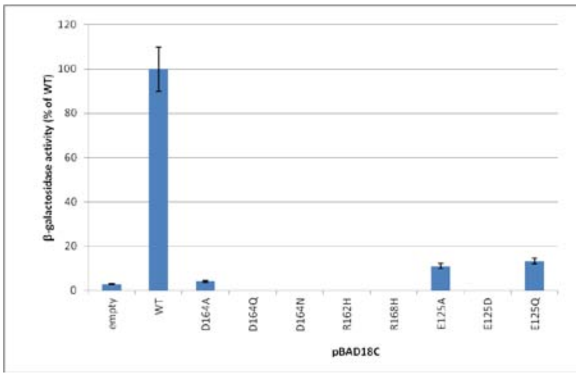

Figure S2, related to Figure 3: ATPase activity of the mixed PspF between A. *cis* (N64, D107 and E108) and all variants used in this study, B. *cis* (N64, D107, E108 and Y126) and E43 variants. Histograms represent the k_{cat} observed for the different variants mixed at equi-molar concentration. Experiment was performed three times independently and the maximal error observed was below 10%.

C. preliminary experiment showing *in vivo* activity of the PspF variants. We used β-galatosidase activity assay of $PspF_{1-275}$ variants E125, R162, D164 and R168 overproduced in MG1655 Δ*pspA*Δ*pspF* strain with chromosomal fusion of *pspAp-lacZ*.

We tested the effect of the substitution of few residues of PspF on its activity *in vivo* using a reporter strain harboring a *pspAp-lacZ* fusion on the chromosome and overproduced PspF variants. As shown in the preliminary results presented in the Figure S2SC, the activities of the variants tested are (when detected) very low and are consistent with the *in vitro* data. It is not possible to readily distinguish from the *in vivo* experiment whether the observed promoter activity reflects a reduction of the specific PspF activity or a reduction in the amount of active protein. As observed

during protein purification, the substitution of a residue located at the interface between subunit often affects the solubility of the protein. Another limitation preventing us from conducting any *in vivo* "mixing" experiment, in the presence of two complementary substitutions, is the probable relative differences in the production of the variants in the same cell at the same time. Hence we did not perform a thorough systematic *in vivo* analysis with all the variants. Rather, *in vitro* methods based on using defined purified soluble proteins measured the effects of these substitutions upon activities.

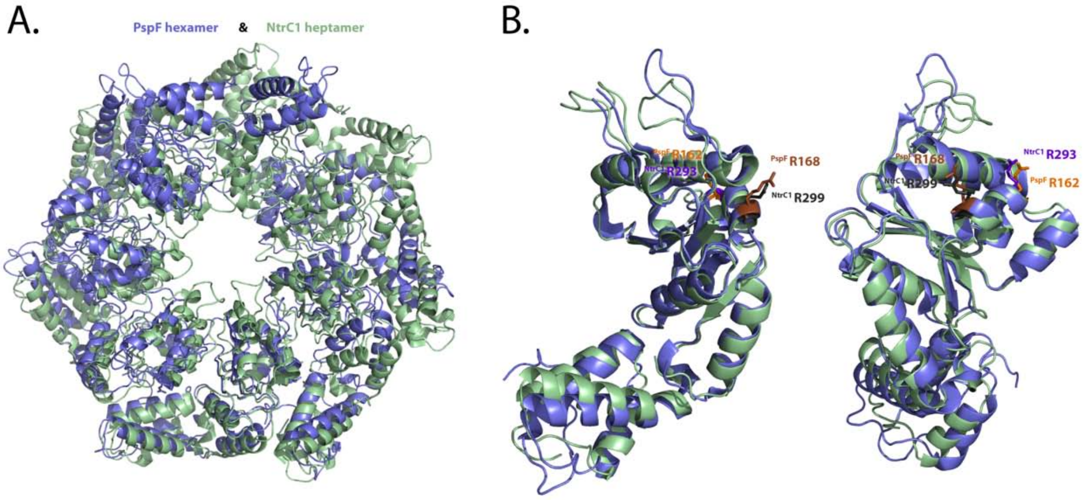

Figure S3, related to Figure 3: Overlayed PspF and NtrC1 structures. A- Overlayed PspF hexamer and NtrC1 heptamer models. B- Overlayed crystal structures of PspF monomer (pdb: 2C9C) and NtrC1 E239A (pdb: 3M0E) with the two arginine residues proposed to be R-fingers residues ^{PspF}R162/^{PspF}R168 and $^{\text{NtrC1}}$R293/$^{\text{NtrC1}}$R299. Figure was generated using PyMol software.

SUPPLEMENTAL REFERENCES

Bordes, P., Wigneshweraraj, S.R., Schumacher, J., Zhang, X., Chaney, M., and Buck, M. (2003). The ATP hydrolyzing transcription activator phage shock protein F of Escherichia coli: identifying a surface that binds sigma 54. Proceedings of the National Academy of Sciences of the United States of America *100*, 2278-2283.

Cannon, W.V., Gallegos, M.T., and Buck, M. (2000). Isomerization of a binary sigma-promoter DNA complex by transcription activators. Nature structural biology *7*, 594-601.

Joly, N., and Buck, M. (2010). Engineered interfaces of an AAA+ ATPase reveal a new nucleotide-dependent coordination mechanism. J Biol Chem *285*, 15178-15186.

Joly, N., Burrows, P.C., and Buck, M. (2008). An intramolecular route for coupling ATPase activity in AAA+ proteins for transcription activation. J Biol Chem *283*, 13725-13735.

Joly, N., Rappas, M., Wigneshweraraj, S.R., Zhang, X., and Buck, M. (2007). Coupling nucleotide hydrolysis to transcription activation performance in a bacterial enhancer binding protein. Molecular microbiology *66*, 583-595.

Joly, N., Schumacher, J., and Buck, M. (2006). Heterogeneous nucleotide occupancy stimulates functionality of phage shock protein F, an AAA+ transcriptional activator. J Biol Chem *281*, 34997-35007.

Lowry, O.H., Rosebrough, N.J., Farr, A.L., and Randall, R.J. (1951). Protein measurement with the Folin phenol reagent. J Biol Chem *193*, 265-275.

Miller, J.H. (1972). Experiments in Molecular Genetics. Cold Spring Harbor Laboratory Press, Cold Spring

Harbor, NY.

Norby, J.G. (1988). Coupled assay of Na+,K+-ATPase activity. Methods Enzymol *156*, 116-119.

Schumacher, J., Zhang, X., Jones, S., Bordes, P., and Buck, M. (2004). ATP-dependent transcriptional activation by bacterial PspF AAA+protein. J Mol Biol *338*, 863-875.

Wigneshweraraj, S.R., Nechaev, S., Bordes, P., Jones, S., Cannon, W., Severinov, K., and Buck, M. (2003). Enhancer-dependent transcription by bacterial RNA polymerase: the beta subunit downstream lobe is used by sigma 54 during open promoter complex formation. Methods Enzymol *370*, 646-657.
